# Multilevel correlates of household anthropometric typologies in Colombian mothers and their infants

**DOI:** 10.1017/gheg.2018.4

**Published:** 2018-04-22

**Authors:** D. C. Parra, L. F. Gomez, L. Iannotti, D. Haire-Joshu, A. K. Sebert Kuhlmann, R. C. Brownson

**Affiliations:** 1 Program of Physical Therapy, Washington University in St. Louis School of Medicine, St. Louis, MO, USA; 2 Rosario University, Bogota, Colombia; 3 Facultad de Medicina, Pontificia Universidad Javeriana, Bogotá, Colombia; 4 Brown School, Washington University in St. Louis, St. Louis, MO 63130, USA; 5 Center For Diabetes Translation Research, Brown School, Washington University in St. Louis, St. Louis, MO 63130, USA; 6 College for Public Health & Social Justice, Saint Louis University, St. Louis, MO 63104, USA; 7 Prevention Research Center in St. Louis, Brown School, Washington University, St. Louis, St. Louis MO 63130, USA; 8 Division of Public Health Sciences and Alvin J. Siteman Cancer Center, Department of Surgery, Washington University School of Medicine, Washington University in St. Louis, St. Louis, MO 63130, USA

**Keywords:** Colombia, Latin America, multilevel models, nutrition transition, obesity

## Abstract

**Background.:**

The aim of this study was to establish the association of maternal, family, and contextual correlates of anthropometric typologies at the household level in Colombia using 2005 Demographic Health Survey (DHS/ENDS) data.

**Methods.:**

Household-level information from mothers 18–49 years old and their children <5 years old was included. Stunting and overweight were assessed for each child. Mothers were classified according to their body mass index. Four anthropometric typologies at the household level were constructed: normal, underweight, overweight, and dual burden. Four three-level [households (*n* = 8598) nested within municipalities (*n* = 226), nested within states (*n* = 32)] hierarchical polytomous logistic models were developed. Household log-odds of belonging to one of the four anthropometric categories, holding ‘normal’ as the reference group, were obtained.

**Results.:**

This study found that anthropometric typologies were associated with maternal and family characteristics of maternal age, parity, maternal education, and wealth index. Higher municipal living conditions index was associated with a lower likelihood of underweight typology and a higher likelihood of overweight typology. Higher population density was associated with a lower likelihood of overweight typology.

**Conclusion.:**

Distal and proximal determinants of the various anthropometric typologies at the household level should be taken into account when framing policies and designing interventions to reduce malnutrition in Colombia.

## Introduction

The nutrition transition was originally described by Popkin as the process in which countries experience a shift in their nutrition and physical activity habits, ultimately resulting in a higher prevalence of diet-related non-communicable diseases such as obesity and diabetes [1]. Popkin proposed three stages depicting the nutrition transition, including receding famine, degenerative disease, and behavioral change. The second stage (degenerative diseases) includes a period where diets are mainly derived from animal source foods with higher intakes of vegetable oils, saturated fats, refined sugar and artificial sweeteners, and higher reliance on production and consumption of foods outside the home, coupled with reduced levels of physical activity. Most countries in Latin America including Colombia, are undergoing the second stage of the nutrition transition, where a large proportion of the nutritional intake comes from edible oils, caloric sweeteners, and animal source foods, combined with a high prevalence of physical inactivity [2]. In this sense, Colombia is still battling both communicable and non-communicable diseases and is faced with the problems of resource allocation and an appropriate design of programs and interventions that will tackle both spectrums of these conditions. This is not an easy task when problems are localized in the same community, let alone in the same household. Thus, it is important to develop appropriate studies capable of classifying the population based on regular surveillance. Currently, many of the anthropometric indicators and measures at the household level focus on the mother–children dyad; but do not take into account information from all of the children under 5. This is of particular relevance for the households of the lowest wealth index category, where the average number of children under 5 years of age is 2.9 *v.* 1.8 for the highest wealth index, and 2.1 for the middle wealth index [3]. For this reason, we developed an anthropometric typology indicator that takes into account information from the mother and all of her children under 5. The accumulating evidence from Latin America on the coexistence of stunting and overweight also known as the dual burden of malnutrition [4], among various members of the same household including mother and children, [5–8] further justifies the use of our anthropometric typology indicator. Prior studies from the region focusing on the dual burden of malnutrition have mostly used a dyad analysis that considers the mother and either the younger or the oldest child under 5 years of age [9, 10].

Moreover, the coexistence of both expressions of malnutrition at the household level suggests the presence of common risk factors and similar causal mechanisms related to the main determinants of the nutrition transition, namely, diet, physical activity, social, and cultural environments. When both undernutrition and overweight coexist in the same contextual factors (family), this provides an indication of related behaviors or risk factors. However, the majority of studies looking at the nutritional outcomes of the population have exclusively focused on the proximal determinants, in particular on family and maternal characteristics. Only a few studies have taken a multilevel approach to understand the influence of social and community characteristics on family nutritional status [11–13]. In Colombia, there is a large variation of the social and economic conditions of the municipalities and states of the country. The national GINI coefficient was estimated at 0.538 in 2014 [14, 15] indicating a serious problem of income misdistribution. Paradoxically, many of the regions with some of the worst social and health indicators are areas of the country receiving a large amount of revenue from multinational corporations exploiting and exploring non-renewable resources such as oil, coal, and gold [16]. It is important to determine if any association exists between social and economic municipal- and state-level indicators and the nutritional status of the population. Since within municipality and state variations are expected both in the outcome and independent indicators, the use of national averages is not recommended. Previous studies conducted in the area of the nutrition transition and the dual burden [13] have mostly used national-level indicators such as GDP [17] but none have focused on exploring specific municipal- and state-level indicators.

This study will provide a novel examination of the correlates of malnutrition at the household, municipal, and state level, exploring its association with various anthropometric typologies at the household level, including the dual burden of malnutrition. To date, most studies in this area have documented national-level prevalence or have focused on a specific region within a country. Therefore, the aim of this study was to establish the association of maternal, family, and contextual correlates of anthropometric typologies at the household level in Colombia.

## Methods

### Data description

This study is a secondary data analysis of the 2005 Demographic Health Survey (DHS/ENDS) from Colombia [3, 18]. DHS/ENDS is a cross-sectional, nationally representative sample of Colombia. The data set included information from children 0–5 years old and their mother aged 18–49 years old (excluding pregnant women and homes with more than one eligible mother). Only cases that had complete information in all family, municipal, and state variables were included; thus, only data from 8300 households were used, excluding a total of 298 households.

Multilevel models allow accounting for the heterogeneity at the state and municipal level and have the unique capacity to address different levels of influence of both upstream and downstream determinants [19]. The increasing recognition of social and physical environmental influences on malnutrition justifies the use of multilevel approaches in this study. Hierarchical non-linear models were selected as they are useful in predicting associations between variables that have an ecological framework with determinants at different levels [20]. GDP growth was selected due to evidence indicating that rapid economic growth is associated with increasing overweight [21]. Other economic development proxy indicators such as living conditions index and royalties, as well as inequality indicators such as unmet basic needs and higher density were selected, given evidence of association with various nutritional outcomes [17, 22].

Municipal and state indicators were obtained from the SIGOT (Geographic Information System for Territorial Planning; in Spanish: *Sistema de Informacion Geografica para la planeacion y el Ordenamiento Territorial*) information system (http://sigotn.igac.gov.co/sigotn/). SIGOT is a publicly available resource with geo-referenced information containing social, health, and economic indicators of the country, the states and the municipalities of Colombia. Institutional Review Board approval for the analyses conducted in this study was not deemed necessary since there were no personal identifiers linking the data to individuals.

### Measures and variables

Weight and height were measured directly by trained staff contracted by the DHS/ENDS survey, using standardized measuring equipment. Weight was measured to the nearest 1.0 kg with participants wearing light clothing and without shoes using a digital weighing scale [23]. Height was measured to the nearest 1.0 cm using a portable stadiometer [24]. Length was measured in children under 2 years old in a prone position and height (in standing position) for children aged 2 years and older.

#### Dependent variables

Nutritional status of children: First, all children 0–5 years from eligible households were classified according to HAZ (height for age *z*-score) to assess stunting (HAZ < −2 s.d.). Overweight and obesity in children was determined using a cut point of >2 s.d. for body mass index – for age *z*-score (BMIz) according to the WHO guidelines [25].

Nutritional status of mother: BMI was determined using WHO cutoff points, underweight (BMI < 18.5 kg/m^2^), overweight (>25 kg/m^2^), and obese (>30 kg/m^2^). The categories of overweight and obese were merged.

Four mutually exclusive categories were developed based on children aged 0–5 years old and maternal nutritional status. To derive these categories, 19 possible combinations of anthropometric status were obtained and were grouped according to the most prevalent distribution within the category, for example, the underweight category is composed of the following potential combinations: (1) underweight mother and all children stunted (0% prevalence), (2) underweight mother and all children normal (3.4% prevalence), (3) underweight mother and at least one stunted child the rest are normal (0.07% prevalence), (4) normal mother and all children stunted (0% prevalence), (5) normal mother and at least one stunted child the remaining are normal (9.3% prevalence). More information about how these categories were constructed, the prevalence of the possible combinations, and how they were grouped can be found elsewhere [26].

- *Normal households*: No stunting or obesity among any of the children and the mother has a normal BMI.
- *Underweight households*: At least one child is stunted, the remaining children can be normal, and the mother is underweight.
- *Overweight households*: At least one child is obese, the remaining children can be normal, and mother is overweight or obese, or normal.
- *Dual burden households*: At least one child is stunted, the remaining children can be normal, and the mother is overweight or obese.

#### Independent variables

Several maternal and family characteristics obtained from DHS/ENDS 2005 were included in this study such as maternal age, maternal education, and wealth index. Additional variables at the municipal and state level included municipal density, municipal living conditions index, municipal royalties (revenue received by the municipality from license agreements with multinational corporations to exploit and explore non-renewable resources such as oil, coal, and gold), state gross domestic product, and state unmet basic needs, and were obtained from the SIGOT data set for the year 2005. The operational definitions and classifications for each of the variables can be found in Table 1.

**Table 1.** Independent variables and operational definitions for maternal, family, municipal, and state characteristics

Variables	Operational definition	Categories	Source
Mother's parity	Number of children that the mother has given birth to (live births)	Three or less More than three	DHS/ENDS
Maternal age	Maternal age in years	18–30 31–39 40–49	DHS/ENDS
Maternal Education level	Maternal school achievement categorized. Variable was also explored as a continuous variable in years	No education Elementary High school or more	DHS/ENDS
Wealth index	The wealth index (WI) proposed by Rutstein and Johnson in 2004, developed for use in DHS was used in this study. WI assesses presence in the household of a range of assets, such as television, type of flooring, water supply, refrigerator, electricity, radio, television, and domestic servant. The index is estimated using principal components and is a continuous variable. Quintile categories of the index already calculated and included in the survey were used, for other analysis they were categorized into three categories	Poor (lowest and second quintiles) Middle (middle quintiles) Rich (fourth and fifth quintiles)	DHS/ENDS
Living conditions index	Quantifies and characterizes living conditions of low-income and not low-income people, also considering poverty. Includes variables related with public utilities, assets and head of household's perception of living conditions	Tertiles: Low 25.57–64.21 Middle 64.44–78.66 High 79.62–91.92	SIGOT
Municipality royalties (regalias)	(Received royalties/total income) × 100 (percentage). Revenue received by the municipality from license agreements with multinational corporations to exploit and explore non-renewable resources such as oil, coal, and gold	Tertiles: Low/middle 5.96–58.44 High 58.95–74.92 Very high 75–96.79	SIGOT
Municipal density	Total number of inhabitants/municipal area in square meters in the urban areas	Tertiles: Low 0.47–40.65 Middle 41.55–195.5 High 199.4−13 687	SIGOT
Unmet basic needs	Percentage of the population with at least one unmet basic need (one or more) deemed necessary to survive in a society. Captures infrastructure conditions and is complemented with indicators of economic dependence and educational assistance	Tertiles: Low 9.2–24.74 Middle 25.03–44.73 High 46.6–79.58	SIGOT
State GDP growth	State gross domestic product growth Indicator that measures the rate of growth in the productivity of the residents belonging to a particular region compared with the previous year	Tertiles: Low 2.34–3.99 Middle 4.49–6.21 High 6.52–23.66	SIGOT

### Data analysis

The DHS/ENDS 2005 and SIGOT data sets were merged using unique municipality and state identifiers in order to obtain one single data set containing all the variables needed for the study. Municipal and state variables were appended to each row of family information. Multi-collinearity between variables was assessed calculating variance inflation factors (VIF) and correlation coefficients. None of the variables included in the model presented correlation coefficients higher than 0.6 or VIF values higher than 3.2. A *p* value of <0.1 was used to determine marginal statistical significance, and a *p* value of <0.05 was used to determine statistical significance. Observations with missing information or implausible values on the outcome, exposure, or any covariate (previously described) were excluded from the analyses.

Four three-level hierarchical polytomous logistic models were developed. These models had household anthropometric typology as the outcome variable, using normal households as the reference category, and predicting the log odds of belonging to any of the remaining typologies, namely, overweight, underweight, and dual burden. The predictors included in the models at level 1 were mother's parity, maternal age, maternal education level, and wealth index; at level 2 were municipal living conditions index, municipal royalties, and municipal density; and at level 3, state unmet basic needs and gross domestic product growth. Slopes at all levels were assumed to be constant to improve statistic efficiency. No cross-level interactions were allowed in the model, meaning that state- and municipal-level variables are allowed to affect only the intercept. This approach was selected because the group average for the dependent variable was assumed to be equal in each group. The random coefficients fixed intercept model also allows for calculating the intra class correlation coefficient (ICC) that estimates the contribution of each variable level to the variance explained. ICC was calculated by dividing the estimated proportion of group-level variance by the estimated total variance.

STATA 9 and HLM version 7.2 were used for analyses. ArcGIS 9.3 software was used to create a series of maps to document the prevalence of the various anthropometric typologies explored in this study (undernutrition, overweight, and dual burden households) by state characteristics including unmet basic needs and gross domestic product.

## Results

Descriptive characteristics of the sample and municipal and state indicators are shown in Table 2. The mean maternal age was 28 years, and the mean number of years of maternal education was 8, which is equivalent to elementary schooling. Overall, maternal parity was 2.5 children. Regarding municipal characteristics, 41% or 93 out of 226 municipalities in the sample had a low living conditions index. Forty-one percent of the municipalities had a high degree of royalties with over 75% of total income originating from them. Forty-three percent of the population was classified as having a middle degree of density.

**Table 2.** Descriptive characteristics of the sample, Colombia 2005

	*n*	Mean or %	s.d./s.e./95% CI
*Maternal and family characteristics*			
Parity (number of children)			
3 or less	7567	81.7	0.0055
>3	1869	1.8	0.0055
Maternal age (years)			
18–30	5675	60.14	0.006
31–39	3196	33.87	0.006
40–49	565	5.99	0.003
Maternal education (years)			
No education	335	0.32	0.002
Elementary	5458	56.9	0.007
High school or more	3643	39.8	0.008
Wealth index (score)			
Poor (lowest and second quintiles)	5018	46.3	0.009
Middle (middle quintiles)	2090	22.2	0.006
Rich (fourth and fifth quintiles)	2328	31.4	0.008
*Municipal characteristics*			
Living conditions index^a^ (%)	226	68.4	12.2
Low 25.57–64.21	93	41%	–
Middle 64.44–78.66	79	35%	–
High 79.62–91.92	54	24%	–
Royalties^b^ (%)	226	67.9	18.7
Low/middle 5.96–58.44	63	28%	–
High 58.95–74.92	70	31%	–
Very high 75–96.79	93	41%	–
Density^c^ (inhabitants/municipal m^2^)	226	385.8	1234.3
Low 0.47–40.65	72	32%	–
Middle 41.55–195.5	97	43%	–
High 199.4–13 687	57	25%	–
*State characteristics*			
Unmet basic needs^d^ (%)	32	38.12	1.89
Low 9.2–24.74	9	28%	–
Middle 25.03–44.73	12	38%	–
High 46.6–79.58	11	34%	–
Gross domestic product growth^e^ (%)	32	5.9	3.8
Low 2.34–3.99	11	34%	–
Middle 4.49–6.21	9	28%	–
High 6.52–23.66	12	38%	–
*Household anthropometric typologies*			
Normal	3574	41.9%	40.4–43.3
Overweight/obese	3289	38.2%	36.5–40.2
Underweight	1158	12.7%	11.7–13.9
Dual burden	577	7.2%	6.2–10.2

a Living conditions index: quantifies and characterizes living conditions of low-income and not low-income people, also considering poverty. Includes variables related with public utilities, assets, and head of household's perception of living conditions.

b Royalties: (received royalties/total income)  ×  100 (percentage).

c Density: total number of inhabitants/municipal area in square meters.

d Unmet basic needs: percentage of the population with at least one unmet basic need (one or more) deemed necessary to survive in a society. Captures infrastructure conditions and is complemented with indicators of economic dependence and educational assistance.

e State gross domestic product: indicator that measures the productivity of the residents belonging to a particular region. Indicates the rate of growth of GDP in percent compared with the previous year.

Overall, the prevalence of stunting (HAZ < −2 s.d.) among children younger than 5 years was 16.3%, 95% CI 15.3–17.1%. The prevalence of overweight/obesity (BMIz > 2 s.d.) among children was 4.5%, 95% CI 3.2–5.1%. The prevalence of underweight (BMI < 18.5 kg/m^2^) among mothers aged 18–49 years old was 4.7%, 95% CI 4.3–5.1%. Meanwhile, the prevalence of overweight/obesity among mothers (BMI ⩾ 25 kg/m^2^) was 41.1% 95% CI 40–42.5%. More information about the distribution of the sample according to the household anthropometric typologies can be found elsewhere [26]. A description of the distribution of household, municipal, and state characteristics by the household anthropometric typology can be seen in Table 3 (included as a Supplementary file).

**Table 3.** Descriptive characteristics of the sample by anthropometric typology, Colombia 2005

	Normal *n* (%)	Underweight *n* (%)	Overweight/obese *n* (%)	Dual burden *n* (%)
*Maternal and family characteristics*				
Parity (number of children)				
3 or less	3049 (85.3)	851 (73.5)	2543 (80.3)	333 (60.5)
>3	525 (14.7)	307 (26.5)	624 (19.7)	217 (39.5)
Maternal age (years)				
18–30	2365 (66.1)	816 (70.4)	1591 (50.0)	300 (54.5)
31–39	1051 (29.4)	293 (25.3)	1323 (42.0)	204 (37.1)
40–49	158 (4.4)	49 (4.2)	253 (8.0)	46 (8.4)
Maternal education (years)				
No education	101 (2.8)	86 (7.4)	76 (2.4)	42 (7.6)
Elementary	1910 (53.4)	778 (67.2)	1802 (56.9)	404 (73.4)
High school or more	1563 (43.7)	294 (25.4)	1289 (40.7)	104 (18.9)
Wealth index (score)				
Poor (lowest and second quintiles)	1814 (50.7)	810 (69.9)	1484 (47.1)	390 (70.9)
Middle (middle quintiles)	819 (22.9)	202 (17.4)	752 (23.7)	103 (18.7)
Rich (fourth and fifth quintiles)	941 (26.3)	146 (12.6)	921 (29.1)	57 (10.4)
*Municipal characteristics*				
Living conditions index^a^ (%)				
Low 25.57–64.21	1044 (29.2)	447 (38.6)	915 (28.9)	218 (39.6)
Middle 64.44–78.66	1217 (34.1)	359 (31.0)	1076 (34.0)	185 (33.6)
High 79.62–91.92	1313 (36.7)	352 (30.4)	1176 (37.1)	147 (26.7)
Royalties^b^ (%)				
Low/middle 5.96–58.44	1198 (34.6)	369 (33.3)	1029 (33.6)	178 (33.3)
High 58.95–74.92	1237 (35.7)	357 (32.2)	1074 (35.0)	162 (30.3)
Very high 75–96.79	1029 (29.7)	382 (34.5)	962 (31.4)	194 (36.3)
Density^c^ (inhabitants/municipal m^2^)				
Low 0.47–40.65	1116 (31.2)	362 (31.3)	1057 (33.4)	191 (34.7)
Middle 41.55–195.5	1101 (30.8)	444 (38.3)	963 (30.4)	214 (38.9)
High 199.4–13 687	1357 (38.0)	352 (30.4)	1147 (36.2)	145 (26.4)
*State characteristics*				
Unmet basic needs^d^ (%)				
Low 9.2–24.74	1246 (34.9)	345 (29.8)	1093 (34.5)	155 (28.1)
Middle 25.03–44.73	1277 (35.7)	371 (32.0)	1210 (38.2)	216 (39.3)
High 46.6–79.58	1051 (29.4)	442 (38.2)	864 (27.3)	179 (32.6)
Gross domestic product growth^e^ (%)				
Low 2.34–3.99	1187 (33.6)	383 (33.3)	1061 (34.0)	193 (35.1)
Middle 4.49–6.21	1256 (35.6)	487 (42.4)	1050 (33.7)	207 (37.7)
High 6.52–23.66	1093 (30.9)	280 (24.4)	1009 (32.3)	149 (27.1)

a Living conditions index: quantifies and characterizes living conditions of low-income and not low-income people, also considering poverty. Includes variables related with public utilities, assets, and head of household's perception of living conditions.

b Royalties: (received royalties/total income) × 100 (percentage).

c Density: total number of inhabitants/municipal area in square meters.

d Unmet basic needs: percentage of the population with at least one unmet basic need (one or more) deemed necessary to survive in a society. Captures infrastructure conditions and is complemented with indicators of economic dependence and educational assistance.

e State gross domestic product: indicator that measures the productivity of the residents belonging to a particular region. Indicates the rate of growth of GDP in percent compared with the previous year.

### Overweight households

Regarding maternal characteristics, maternal education and maternal age were associated with the overweight household typology in all three models, and remained significant even after including municipal- and state-level indicators in the model. Maternal age categories of 31–39 years (OR 1.9, 95% CI 1.6–2.0) and 40–49 years (OR 2.3, 95% CI 1.8–2.9) were positively associated with the overweight typology compared with normal households. The poor (OR 0.8, 95% CI 0.6–0.8) category of wealth index was negatively associated with the likelihood of being classified in the overweight category *v.* normal category. Regarding municipal indicators, living conditions index and municipal density were associated with the prevalence of overweight households even after adjusting for state-level variables in the model. High (OR 1.5, 95% CI 1.1–2.0) living conditions index was associated with higher odds of overweight *v.* normal households. Municipalities with high levels of density (OR 0.6, 95% CI 0.4–0.8) were associated with lower odds of overweight. None of the state-level indicators were statistically associated with the overweight typology (Table 4).

**Table 4.** Multilevel model – maternal, family, municipal, and state correlates of anthropometric typologies in 2005 (families *n* = 8598, municipalities *n* = 226, state *n* = 32)

	Overweight typology^a^			Underweight typology^b^			Dual burden typology^c^		
	Model 1 OR (CI)-*p* value	Model 2 OR (CI)-*p* value	Model 3 OR (CI)-*p* value	Model OR (CI)-*p* value	Model 2 OR (CI)-*p* value	Model 3 OR (CI)-*p* value	Model 1 OR (CI)-*p* value	Model2 OR (CI)-*p* value	Model 3 OR (CI)-*p* value
*Maternal and family indicators*									
Parity									
<3 children	Ref	Ref	Ref	Ref	Ref	Ref	Ref	Ref	Ref
>3 children	1.0 (0.8–1.2)-0.08	1.0 (0.8–1.2)-0.08	1.0 (0.8–1.2)-0.08	**1.8 (1.5–2.1)-<0.001**	**1.8 (1.4–2.2)-<0.001**	**1.8 (1.4–2.2)-<0.001**	**2.3 (1.8–2.8)-<0.001**	**2.3 (1.8–2.9)-<0.001**	**2.3 (1.8–2.9)-<0.001**
Education									
High school or more	Ref	Ref	Ref	Ref	Ref	Ref	Ref	Ref	Ref
Elementary	1.2 (1.0–1.3)-0.057	**1.2 (1.1–1.3)-0.003**	1.2 (1.0–1.3)-0.04	**1.5 (1.3–1.9)-<0.001**	**1.5 (1.3–1.8)-<0.001**	**1.6 (1.3–1.8)-<0.001**	**2.0 (1.6–2.5)-<0.001**	**2.0 (1.5–2.6)-<0.001**	**2.0 (1.5–2.6)-<0.001**
No education	0.9 (0.6–1.3)-0.09	0.9 (0.6–1.2)-0.09	0.90 (0.6–1.2)-0.09	**2.4 (1.6–3.4)-<0.001**	**2.4 (1.7–3.4)-<0.001**	**2.4 (1.6–3.3)-<0.001**	**2.6 (1.6–4.0)-<0.001**	**2.5 (1.5–3.9)-<0.001**	**2.5 (1.5–3.9)-<0.001**
Age									
18–30 years	Ref	Ref	Ref	Ref	Ref	Ref	Ref	Ref	Ref
31–39 years	**1.9 (1.6–2.1)-<0.001**	**1.9 (1.6–2.0)-<0.001**	**1.9 (1.6–2.0)-<0.001**	**0.7 (0.6–0.8)-0.002**	**0.7 (0.5–0.8)-0.002**	**0.7 (0.5–0.8)-0.002**	1.2 (0.9–1.4)-0.07	1.2 (0.9–1.4)-0.07	1.2 (0.9–1.4)-0.07
40–49 years	**2.3 (1.8–2.9)-<0.001**	**2.3 (1.8–2.9)-<0.001**	**2.3 (1.8–2.9)-<0.001**	**0.5 (0.4–0.7)-<0.001**	**0.5 (0.3–0.8)-<0.001**	**0.5 (0.3–0.8)-<0.001**	1.3 (0.9–1.8)-0.09	1.3 (0.8–1.8)-0.09	1.3 (0.8–1.8)-0.07
Wealth index									
Rich	Ref	Ref	Ref	Ref	Ref	Ref	Ref	Ref	Ref
Middle	0.9 (0.8–1.0)-0.09	0.9 (0.7–1.0)-0.08	0.9 (0.7–1.0)-0.08	1.3 (1.0–1.7)-0.09	1.3 (1.0–1.7)-0.09	1.3 (1.0–1.7)-0.09	**1.7 (1.2–2.3)-<0.001**	**1.7 (1.2–2.4)-<0.001**	**1.7 (1.2–2.3)-<0.001**
Poor	**0.8 (0.6–0.9)-0.002**	**0.8 (0.6–0.8)-0.002**	**0.8 (0.6–0.8)-0.002**	**2.0 (1.6–2.5)-<0.001**	**2.0 (1.5–2.5)-<0.001**	**1.9 (1.5–2.5)-<0.001**	**2.2 (1.6–2.8)-<0.001**	**2.0 (1.4–2.9)-<0.001**	**2.1 (1.4–2.9)-<0.001**
*Municipal indicators*									
Living conditions index^d^									
Low (25.57–65.76)		Ref	Ref		Ref	Ref		Ref	Ref
Middle (65.92–80.4)		1.1 (0.9–1.3)-0.09	1.1 (0.9–1.3)-0.09		0.8 (0.6–1.0)-09	0.9 (0.6–1.0)-0.09		0.9 (0.6–1.2)-0.09	0.9 (0.6–1.2)-0.09
High (80.58–91.92)		**1.5 (1.1–2.0)-<0.001**	**1.5 (1.1–2.0)-<0.001**		0.9 (0.5–1.3)-0.08	0.9 (0.6–1.4)-0.08		0.9 (0.4–1.6)-0.09	0.8 (0.4–1.6)-0.09
Royalties^e^									
Low/middle (5.9–58.35)		Ref	Ref		Ref	Ref		Ref	Ref
High (58.36–74.31)		1.1 (0.8–1.2)-0.09	1.1 (0.8–1.2)-0.08		0.8 (0.6–1.0)-0.09	**0.8 (0.5–0.9)-<0.001**		0.8 (0.5–1.0)-0.07	0.7 (0.4–1.0)-0.012
Very high (74.92–96.79)		1.1 (0.9–1.4)-0.08	1.1 (0.9–1.4)-0.09		0.9 (0.7–1.2)-0.09	0.9 (0.6–1.1)-0.09		0.9 (0.6–1.2)-0.09	0.8 (0.5–1.2)-0.09
Density^f^									
Low (0.47–42.19)		Ref	Ref		Ref	Ref		Ref	Ref
Middle (42.36–217.12)		0.9 (0.7–1.1)-0.073	0.9 (0.7–1.1)-0.08		1.3 (1.0–1.5)-0.09	1.3 (1.0–1.6)-0.09		1.2 (0.9–1.6)-0.09	1.3 (0.9–1.7)-0.015
High (228.94–13 687.06)		**0.6 (0.4–0.8)-<0.001**	**0.6 (0.4–0.8)-<0.001**		1.1 (0.7–1.6)-0.09	1.0 (0.7–1.6)-0.09		0.9 (0.5–1.5)-0.09	0.9 (0.5–1.7)-0.09
*State indicators*									
Unmeet basic needs^g^									
Low (9.2–24.74)			Ref			Ref			Ref
Middle (25.03–44.73)			1.0 (0.7–1.3)-0.09			1.0 (0.7–1.4)-0.07			1.4 (0.8–2.2)-0.09
High (46.6–79.58)			0.9 (0.7–1.3)-0.09			1.1 (0.8–1.6)-0.08			2.2 (0.7–2.0)-0.08
Gross domestic product growth^h^									
Low (2.34–3.99)			Ref			Ref			Ref
Middle (4.49–6.21)			1.0 (0.8–1.2)-0.06			1.1 (0.8–1.4)-0.08			1.0 (0.7–1.4)-0.06
High (6.52–23.66)			1.0 (0.7–1.4)-0.07			0.8 (0.5–1.2)-0.08			0.9 (0.5–1.6)-0.07

Bolded odds ratios indicate significance of *p* < 0.05.

a Underweight households: at least one child in stunting (HAZ < −2 s.d.), the rest of the children can be normal and mother underweight by BMI (BMI < 18. 5 kg/m^2^).

b Overweight households: at least one child with overweight according to BMIz score (BMIz > −2 s.d.), the rest of the children can be normal and mother overweight or obese by BMI (BMI > 25 or >30 kg/m^2^), or normal.

c Dual burden households: at least one child in stunting (HAZ < −2 s.d.), the rest of the children can be normal and mother overweight or obese by BMI (BMI > 25 or >30 kg/m^2^).

d Living conditions index: quantifies and characterizes living conditions of low-income and not low-income people, also considering poverty. Includes variables related with public utilities, assets, and head of household's perception of living conditions.

e Royalties: (received royalties/total income) × 100 (percentage).

f Density: total number of inhabitants/municipal area in square meters.

g Unmet basic needs: percentage of the population with at least one unmet basic need (one or more) deemed necessary to survive in a society. Captures infrastructure conditions and is complemented with indicators of economic dependence and educational assistance.

h State gross domestic product: indicator that measures the productivity of the residents belonging to a particular region. Indicates the rate of growth of GDP in percent compared with the previous year.

Model 1: includes maternal and family characteristics.

Model 2: includes maternal and family characteristics as well as municipal indicators.

Model 3: final model, fully adjusted. Includes maternal and family characteristics as well as municipal and state variables.

### Underweight households

Regarding maternal and family characteristics, parity, maternal education, maternal age, and wealth index were associated with the underweight anthropometric typology in all three models, and remained significant even after including municipal- and state-level indicators in the model. Maternal parity of more than three children (OR 1.8, 95% CI 1.4–2.2) was associated with higher odds of underweight. Having maternal elementary education and no education were associated with a larger likelihood of underweight compared with normal households. Maternal age categories of 31–39 years (OR 0.7, 95% CI 0.5–0.8) and 40–49 years (OR 0.5, 95% CI 0.3–0.8) had lower odds of underweight compared with normal households. The poor (OR 1.9, 95% CI 1.5–2.5) category of wealth index was positively associated with the likelihood of being classified in the underweight typology *v.* normal. Regarding municipal indicators, living conditions index, royalties, and municipal density were associated with the prevalence of underweight households even after including state-level variables in the model. Municipalities in the middle range for royalties (OR 0.8, 95% CI 0.5–0.9) had significantly lower odds of being classified in the underweight typology *v.* normal. None of the state-level indicators were statistically associated with the underweight typology (Table 4).

### Dual burden households

Regarding maternal and family characteristics, parity, maternal education, maternal age, and wealth index were associated with the dual burden anthropometric typology in all three models, and remained significant even after including municipal- and state-level indicators in the model. Maternal parity of more than three children (OR 2.3, 95% CI 1.8–2.9) was associated with a higher likelihood of dual burden. Having maternal elementary education (OR 2.0, 95% CI 1.5–2.6) and no formal education (OR 2.5, 95% CI 1.5–3.9) were associated with higher odds of dual burden compared with normal households. The middle (OR 1.7, 95% CI 1.2–2.3) and poor (OR 2.1, 95% CI 1.4–2.9) categories of wealth index were positively associated with the likelihood of being classified as a dual burden household *v.* normal. Only the municipal indicators of royalties and density were correlated with the dual burden typology, although this association was marginally significant. Municipalities in the middle range for royalties had a significantly lower likelihood of being classified as a dual burden *v.* normal households. Municipalities with middle levels of density were associated with a higher likelihood of dual burden *v.* normal households. None of the state-level indicators were statistically associated with the dual burden typology (Table 4).

### Variance components

Twenty-nine percent of the variance within families in the category of underweight is explained by family variables, 15% by municipal variables, and 1.6% by state variables. Regarding the category of overweight/obesity, 26% of the variance within families is explained by family variables, 6.2% is explained by municipal variables, and only 2% by state variables. Seventeen percent of the variance within families in the category of dual burden is explained by family variables, 4.2% is explained by municipal variables, and 2.7% by state variables.

## Discussion

This study found that several household (maternal and family) characteristics are associated with various anthropometric typologies at the household level, namely, overweight, underweight, and the dual burden of malnutrition. Municipal indicators were associated to a lesser degree, and only three of them were found to be significant in the fully adjusted models; high living conditions index and high density were associated with the overweight typology; and middle royalties were associated with the underweight typology. No associations were detected for state indicators.

Higher maternal parity was positively associated with both the underweight and the dual burden typology; this is a seemingly paradoxical association given that a dual burden household includes an overweight mother. However, there has been evidence showing that high parity is associated with both maternal underweight and overweight and this relationship seems to be mediated by family income level and other economic characteristics of the region [27–29]. The fact that parity was associated with both the underweight and dual burden typologies, independent of wealth index and maternal education, suggest that there are other underlying factors at play, which were not captured by this study, and the topic warrants further exploration.

The average number of years for maternal education was 8 years, which is barely beyond elementary school. Non-formal and only elementary maternal education was associated with a higher prevalence of overweight, underweight, and dual burden households. Given the importance of maternal education in improving the nutritional status of the family [30], it is important to improve the degree and quality of education among Colombian women [12]. In addition, the positive direction of association for the overweight typology with elementary maternal education and the negative direction of association with wealth index show that both variables are capturing different aspects and clusters of risk factors. Both wealth index and maternal education should be considered when developing studies of family nutritional status as well as when developing programs and policies to address malnutrition.

A high index of municipal living conditions was positively correlated with the prevalence of overweight households. This finding highlights the fact that Colombia is in the midst of a nutrition transition where overweight is still more prevalent among higher socioeconomic conditions [26]. Municipal royalties were correlated with the underweight nutrition typology. A high range of municipal royalties (58.9–74.9% of municipal income derived from royalties) was associated with a lower likelihood of underweight. This association could point to a good use of economic resources by the municipalities; however, due to the cross-sectional nature of this study, no conclusions can be drawn in this regard and more detailed research on this topic is granted.

Finally, a higher level of municipal density (228.94–13 687.06 inhabitants/municipal m^2^) was associated with a lower likelihood of overweight households. This could perhaps point to the fact that highly dense areas are more walkable and they imply more energy expenditure [31]. Density can act as a proxy for urbanization level, in this sense, higher density may equal highly urbanized areas and thus greater opportunities for physical activity; in particular transportation related, which may in turn reduce the likelihood of overweight. However, these are only hypotheses, and again due to the cross-sectional nature of this study, more research is needed to be able to make final conclusions.

Some limitations from this study should be noted. First, the use of secondary data analysis limits the reach of our results due to issues such as social desirability of responses, measurement errors, and the inability to obtain additional information from the respondents. In addition, there is also the possibility of misalignment with the original hypotheses and thus the design of study as it relates to the explorations from this analysis. Secondary analysis of large data sets can present challenges related to the lack of clarity about the organization of the variables; this limitation was reduced by working closely with the statisticians who developed the actual surveys and the sampling strategy. In addition, the information regarding the municipal and state indicators was obtained from a different data set that was not linked to the household information from ENSIN, and it was merged solely based on the information from the reported municipality or state of residence and as such, misclassification could have occurred. In most cases, only the middle categories of all municipal indicators were associated or borderline associated with both the underweight and the dual burden typologies, if at all, with no associations for the highest categories. Unfortunately we are unable to specifically pinpoint as to why this might have been the case. This may be due in part to the quality of the data, which may have lacked specificity or that the number of observations limited the power to detect meaningful differences. In addition, since our specific set of anthropometric typologies in some cases combines households with both overweight and normal weight mothers, as in the overweight household category, the sensitivity of analyses to capture associations could have been affected. Finally, this is a cross-sectional analysis, and no conclusions about temporality of associations can be drawn.

Even with these limitations, results from this study show that it is important to explore nutritional outcomes with the use of multilevel models that allow for including important contextual variables of the municipality as well as family and maternal correlates while exploring a household-level outcome variable such as anthropometric typologies in the case of this study. The addition of state-level variables and most of the municipal variables did not contribute in explaining differences in the anthropometric typologies; however, this does not mean that state or municipal indicators are not important and could still have an important role on the clustering of responses; however, further research on this topic should be carried out.

This paper contributes to the scarce but growing evidence about the nutrition transition and the dual burden of malnutrition in Colombia, and it provides important information of public health relevance regarding the various correlates of previously identified nutrition typologies in the country [26]. Evidence from Colombia and other middle-income countries highlights the key role of maternal education and income (proximal determinants) for children's adequate nutritional status.
